# Novel Mg-ion conductive oxide of μ-cordierite Mg_0.6_Al_1.2_Si_1.8_O_6_

**DOI:** 10.1080/14686996.2020.1730237

**Published:** 2020-03-03

**Authors:** Hayami Takeda, Koki Nakano, Naoto Tanibata, Masanobu Nakayama

**Affiliations:** a Department of Advanced Ceramics, Nagoya Institute of Technology, Nagoya, Japan; b Elements Strategy Initiative for Catalysts and Batteries (ESICB), Kyoto University, Kyoto, Japan; c Frontier Research Institute for Materials Science (FRIMS), Nagoya Institute of Technology, Nagoya, Japan; d Center for Materials Research by Information Integration (CMI2), Research and Services Division of Materials Data and Integrated System (Madis), National Institute for Materials Science (NIMS), Tsukuba, Japan

**Keywords:** Mg ion batteries, solid electrolytes, high-throughput materials search, AC impedance spectra, density functional theory, 207 Fuel cells / Batteries / Super capacitors, 401 1st principle calculations, 107 Glass and ceramic materials

## Abstract

Solid electrolytes with high Mg-ion conductivity are required to develop solid-state Mg-ion batteries. The migration energies of the Mg^2+^ ions of 5,576 Mg compounds tabulated from the inorganic crystal structure database (ICSD) were evaluated via high-throughput calculations. Among the computational results, we focused on the Mg^2+^ ion diffusion in Mg_0.6_Al_1.2_ Si_1.8_O_6_, as this material showed a relatively low migration energy for Mg^2+^ and was composed solely of ubiquitous elements. Furthermore, first-principles molecular dynamics calculations confirmed a single-phase Mg^2+^ ion conductor. The bulk material with a single Mg_0.6_Al_1.2_Si_1.8_O_6_ phase was successfully prepared using the sol-gel method. The relative density of the sample was 81%. AC impedance measurements indicated an electrical conductivity of 1.6 × 10^−6^ Scm^−1^ at 500°C. The activation energy was 1.32 eV, which is comparable to that of monoclinic-type Mg_0.5_Zr_2_(PO_4_)_3_.

## GRAPHICAL ABSTRACT

**Figure:**
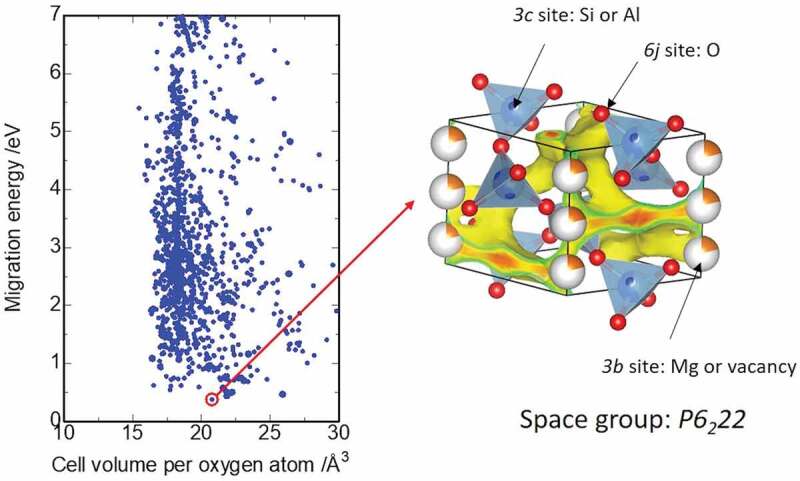

## 1. Introduction

Li-ion batteries are widely used in portable electronic devices, such as mobile phones and laptop computers. Recently, electric vehicles have become more widely used, as part of efforts to address environmental and energy issues [1,2]. Therefore, Li-ion batteries are attracting attention as second batteries for these electric vehicles. However, there are two major drawbacks associated with these batteries [3]: (1) their short driving range owing to their low energy density and (2) the risk of ignition owing to the use of flammable electrolytes

The use of Mg-ion batteries is one way of solving the first drawback, because of their large capacity [4]. Mg is divalent and it can carry two electrons, and would thus be expected to double the energy density of Li-ion batteries [5,6]. The second drawback can be overcome by developing all-solid-state batteries using nonflammable solid electrolytes. Solid electrolytes with high Mg-ion conductivities are thus required to develop all solid-state Mg-ion batteries. Several Mg-containing materials have been proposed as candidates for inorganic solid electrolytes [5,7–18]. In addition to the above, a stable supply of elemental resources is needed; Mg is more abundant than Li, and can meet the increasing need for large-scale batteries. Nevertheless, the rather slow diffusivity of Mg^2+^ ions in oxides significantly lowers their charge–discharge rate performance at room temperature. Spinel-type selenides and sulfides have exhibited sufficiently fast ionic conductivity with Mg^2+^ [5]. Nevertheless, the use of stable oxides for solid electrolytes would be attractive in terms of abundance and phase stability [19,20], as sulfides are unstable in air [21]. Therefore, it is necessary to find new oxides with fast Mg^2+^ ion conductivity. In this respect, we recently performed a high-throughput exhaustive search for fast ion conductors via automated material simulations, using the force field technique and percolation theory [22]. We demonstrated the effectiveness of novel high-rate electrodes comprising Na_2_V_3_O_7_ as cathodes for Na-ion batteries [23].

Similarly, in this study, we performed the high-throughput calculations mentioned above for ~ 6,903 Mg-O-containing compounds listed in the inorganic crystal structure database (ICSD) [24] and selected μ-cordierite Mg_0.6_Al_1.2_Si_1.8_O_6_ [25] as the candidate oxide compound for solid electrolytes. The conductivity of Mg^2+^ was evaluated through both accurate first-principles molecular dynamics (FPMD) calculations, and experimental alternating current (AC) impedance measurements.

## 2. Molecular dynamics computations

**Figure 1.**
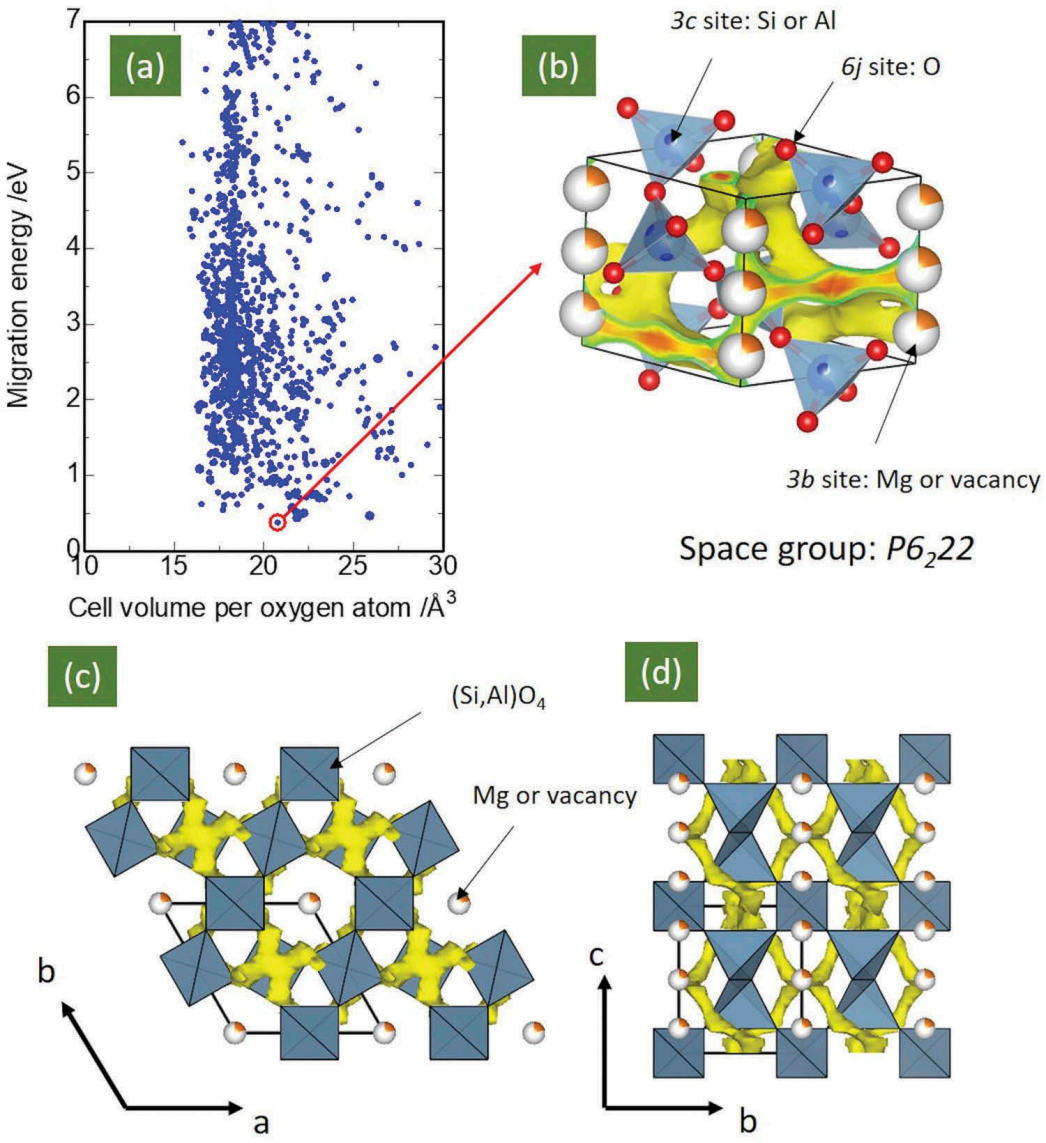
Panel (a) Calculated migration energies of 5,576 inorganic solid-state samples containing both Mg and O, extracted from the inorganic crystal structure dataset (ICSD) [19] using bond valence based force field (BVFF) [21] potential calculations and a percolation algorithm. The red open circle in panel (a) corresponds to Mg_0.6_Al_1.2_Si_1.8_O_6_ (ICSD #24,898). Panels (b)–(d) show the visualization of the isosurface with various potential energies obtained from BVFF calculations involving Mg_0.6_Al_1.2_Si_1.8_O_6_. The light-blue polyhedra and orange/white spheres represent (Si/Al)O_4_ tetrahedra and Mg/vacancies, respectively. Yellow-colored isosurfaces indicate the migration pathways of Mg ions in terms of their potential energy distribution. Note that the migration energy is the difference between the maximum and minimum of the potential energy in the migration pathway

Migration energies were roughly evaluated for 5,576 samples (blue symbols in Figure 1) using the bond valence-based force field (BVFF) [26] potential calculation and percolation algorithm [22]. In detail, the real space voxel division (0.016 Å^3^) for the lattice was made, and the potential energies of Mg^2+^ ions at each voxel were computed using the BVFF. The migration energy was determined when the lower-potential voxel had percolated throughout the lattice. Details of the method are described further in the literature [26]. Density-functional-theory-based (DFT) FPMD simulations were performed to investigate diffusion of Mg^2+^ in Mg_0.6_Al_1.2_Si_1.8_O_6_ systems. The Vienna *ab initio* simulation package (VASP) [27–30] was used with the projector augmented-wave (PAW) method [31,32] and a plane-wave basis set. We used a generalized gradient approximation (GGA)-type exchange-correlation function developed by Perdew, Burke, and Ernzernhof, and modified it for solid materials (PBEsol) [33]. The cutoff energy was set to 350 eV and a 1 × 1 × 1 *k*-point grid (only Γ point) was employed to reduce the computational cost. The time step was set to 1 fs, and our FPMD simulations were carried out in the NVT canonical ensemble using a Nosé thermostat [34], across a temperature range of 1673 to 1973 K, for > 50 ps.

## 3. Experimental measurements

Mg_0.6_Al_1.2_Si_1.8_O_6_ was synthesized by the sol-gel method [35–39]. Al(NO_3_)_3_･9H_2_O (Wako Pure Chemical Industry), Mg(CH_3_COO)_2_･4H_2_O (Soekawa Chem. Co., Ltd.), and tetraethyl orthosilicate (Sigma – Aldrich Co. LLC.), were used as the starting materials. Stoichiometric amounts of Al(NO_3_)_3_･9H_2_O and Mg(CH_3_COO)_2_･4H_2_O were dissolved in ethylene glycol at room temperature. Then, the stoichiometric amount of tetraethyl orthosilicate was added and the mixture was stirred via ultrasonication. The solution gelled at 55°C. The obtained gel was dried at 100°C. The dried powder was then calcinated at 700°C for 2 h, and was subsequently pelletized under a pressure of 38 MPa. The pellet was sintered at 1000°C for 2 h. The sample size was approximately 8 mm in diameter and 0.8 mm in thickness. The relative density was evaluated from the weight and volume of the sample.

The crystal phase of the sample was characterized via x-ray diffraction (XRD) using a MiniFlex 600 diffractometer (Rigaku, Japan) with Cu-Kα radiation. The micromorphology of the sample was examined using a scanning electron microscope (SEM, JCM-6360LVS, JEOL, Japan). The thermal stability was investigated using thermogravimetric and differential thermal analysis (TG-DTA, EXSTAR 6000; Seiko Instruments Inc, Japan), in a temperature range from room temperature to 1000°C. The ionic conductivity of the bulk sample was measured through AC impedance spectroscopy. Impedance spectroscopy was performed using the sintered pellets. Both faces were polished with 1200-grit abrasive papers and sputtered with gold, so that they could be used as electrodes. The complex impedance was measured using an impedance analyzer (Logic VMP 300; Biologic, France) in the temperature range from 450 to 600°C, at frequencies ranging from 0.1 Hz to 0.5 MHz and a voltage of 1 V, in flowing argon (Ar). To determine the exact ionic conductivity in the AC impedance measurements, DC measurements were also performed.

## 4. Results and discussion

### 4.1. Computational results

Figure 1(a) presents the migration energies of 5,576 samples (blue symbols) determined through BVFF potential calculations and the percolation algorithm [22,26]. Note that ~ 20% of the oxides registered in the database (6,903 samples) were discarded, mainly due to i) the unavailability of force field (FF) parameter sets; and ii) the existence of too short bonds in the lattice, which made the valence state assignment difficult via the bond valence (BV) approach. The experimentally determined crystal structures often contained partially occupied defect sites or splitting sites, resulting in their bonds being too short. Among the 5,576 calculated samples, ~ 24% consisted of only group 2–5 atoms; lanthanides; or Zn, Al, Ga, In, Si, Ge, Sn, P, or S elements, in addition to Mg and O (periods of 7 and later were discarded). These compounds do not contain open-shell transition metal ions, so pure ionic conductivity would be expected. The open red circle in Figure 1(a) corresponds to the μ-cordierite Mg_0.6_Al_1.2_Si_1.8_O_6_ compound. This material was chosen as the candidate solid electrolyte for Mg-ion batteries owing to its significantly low migration energy (~ 0.4 eV). Moreover, this compound consists of abundant s- or p-block metallic elements, thus preventing electronic conduction. Panels (b) through (d) display the crystal structure of Mg_0.6_Al_1.2_Si_1.8_O_6_, and show visualizations of the isosurface with various potential energies obtained from BVFF calculations. As determined earlier [25], this material belongs to the space group *P6_2_22*. O ions occupy 6 j sites, and Si or Al ions are located at tetrahedral 3 c sites. The SiO_4_ or AlO_4_ tetrahedra share their vertices, forming a one-dimensional tunnel along the c axis (Panel (c)). Mg ions occupy 3b sites at the center of the tunnel. However, the migration path indicated in the potential isosurface is a three-dimensional path with a spiral-like network along the c axis. The Mg ions avoided positions at the center of the tunnel along the c axis when the lowest-energy migration path is considered (panels (c) and (d)). However, the energy difference between the site at the center of the tunnel and the lowest-energy migration path was less than 0.5 eV, according to BVFF calculations.

**Figure 2.**
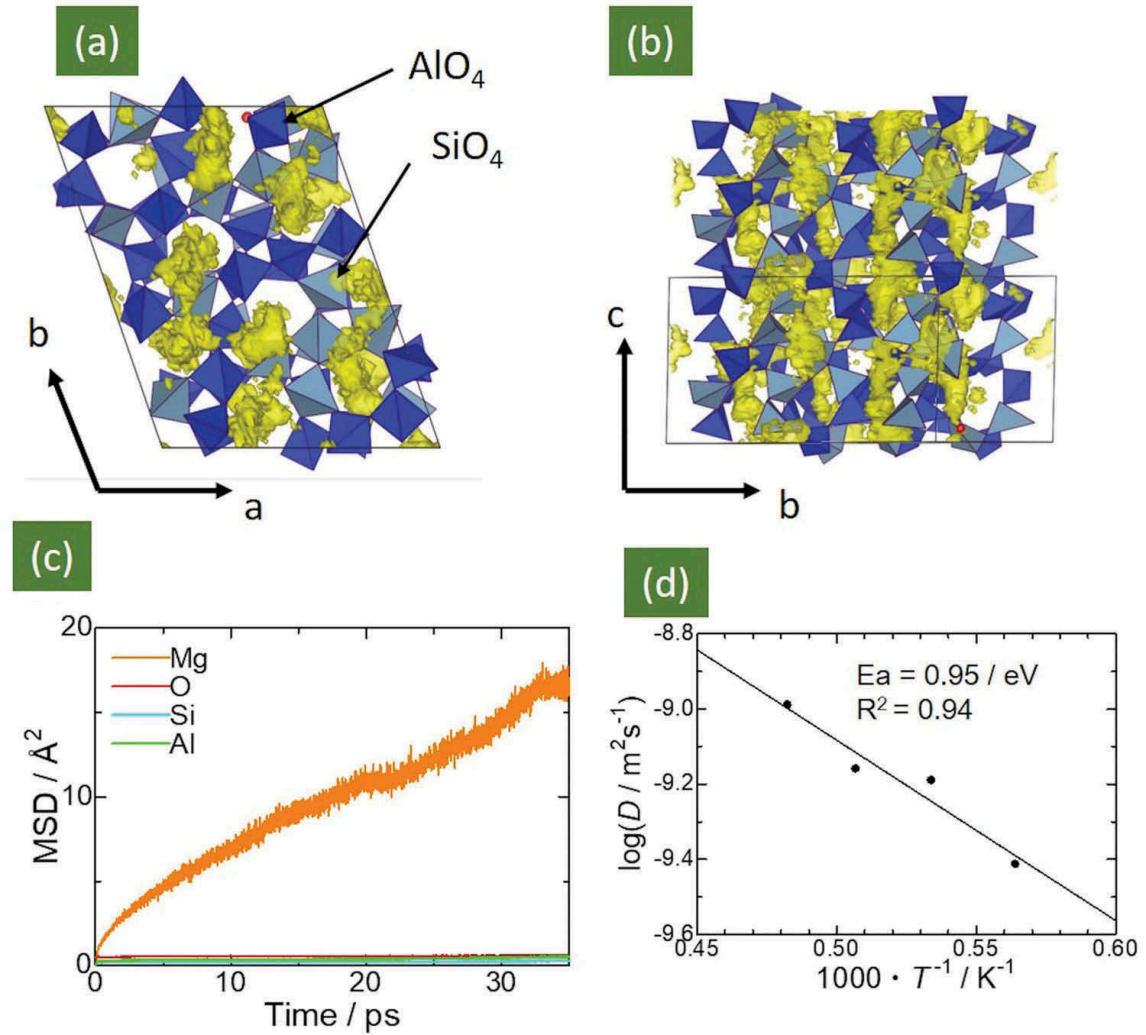
Panels (a) and (b) present population density of Mg ions in Mg_0.6_Al_1.2_Si_1.8_O_6_, derived from 50 ps FPMD calculations. Light- and dark-blue tetrahedra represent SiO_4_ and AlO_4_ units, respectively. Yellow-colored isosurface represents population density of Mg ions. Panel (c) shows MSD plots of Mg, Al, Si, and O trajectories in Mg_0.6_Al_1.2_Si_1.8_O_6_ at 1723 K. Panel (d) presents the temperature-dependent Mg-ion diffusion coefficient in the Arrhenius plot. The calculated activation energy (migration energy) was 0.95 eV

Figure 2 summarizes the FPMD simulation results for the Mg_0.6_Al_1.2_Si_1.8_O_6_ compound. Panels (a) and (b) display the Mg ion population densities during FPMD simulations at 1978 K. The Mg ions were mainly distributed around the tunnel sites along the c axis, and the intertunnel jump was also visible, as indicated in the BVFF calculations (Figure 1(c,d)), Therefore, a three-dimensional diffusion pathway for Mg ions was formed in Mg_0.6_Al_1.2_Si_1.8_O_6_. Panel (c) displays the mean square displacement (MSD) of all of the elements as a function of the simulation time at 1873 K. The MSD profile of Mg ions shows a linear increase with the simulation time, indicating that the Mg ions hopped among these sites and diffused over the lattice. However, the MSD profiles of Si, Al, and O ions, were constant (< 1 Å^2^), indicating thermal vibration. The self-diffusion coefficient, D, for Mg was estimated from the slope of the MSD plots, and the estimated self-diffusion coefficients were summarized in the Arrhenius plot in Figure 2(d). The activation energy for Mg-ion diffusion was 0.95 eV, which represents from a large deviation from the value obtained via BVFF calculations. The possible reasons for this deviation are (i) ignoring Mg–Mg interactions in the BVFF + percolation approach [22,26], (ii) choosing inappropriate FF parameters, and (iii) temperature differences between the BVFF + percolation approach (0 K) and FPMD calculations (> 1673 K).

The extrapolated Mg-ion conductivity estimated using the Nernst–Einstein relationship [40] at 500°C was ~ 6 × 10^−5^ S cm^−1^; this value is comparable to the conductivity of Mg_0.5_Zr_2_(PO_4_)_3_, one of the representative fast Mg-ion conductors (See Table 1).

**Table 1.** Ionic conductivities of Mg^2+^ at 500°C in different compounds

Composition	Ionic conductivity / Scm^−1^ (500°C)	References
Mg_0.6_Al_1.2_Si_1.8_O_6_(experimental)	2.3 × 10^−6^	
Mg_0.6_Al_1.2_Si_1.8_O_6_(computational)	5.6 × 10^−5^	
MgZr_4_P_6_O_24_	1.6 × 10^−6^	[8]
Mg_0.7_(Zr_0.85_Nb_0.15_)_4_(PO_4_)_6_	1.6 × 10^−4^	[7]

### 4.2.. Crystal phase, microstructure, and thermal stability of the sample

**Figure 3.**
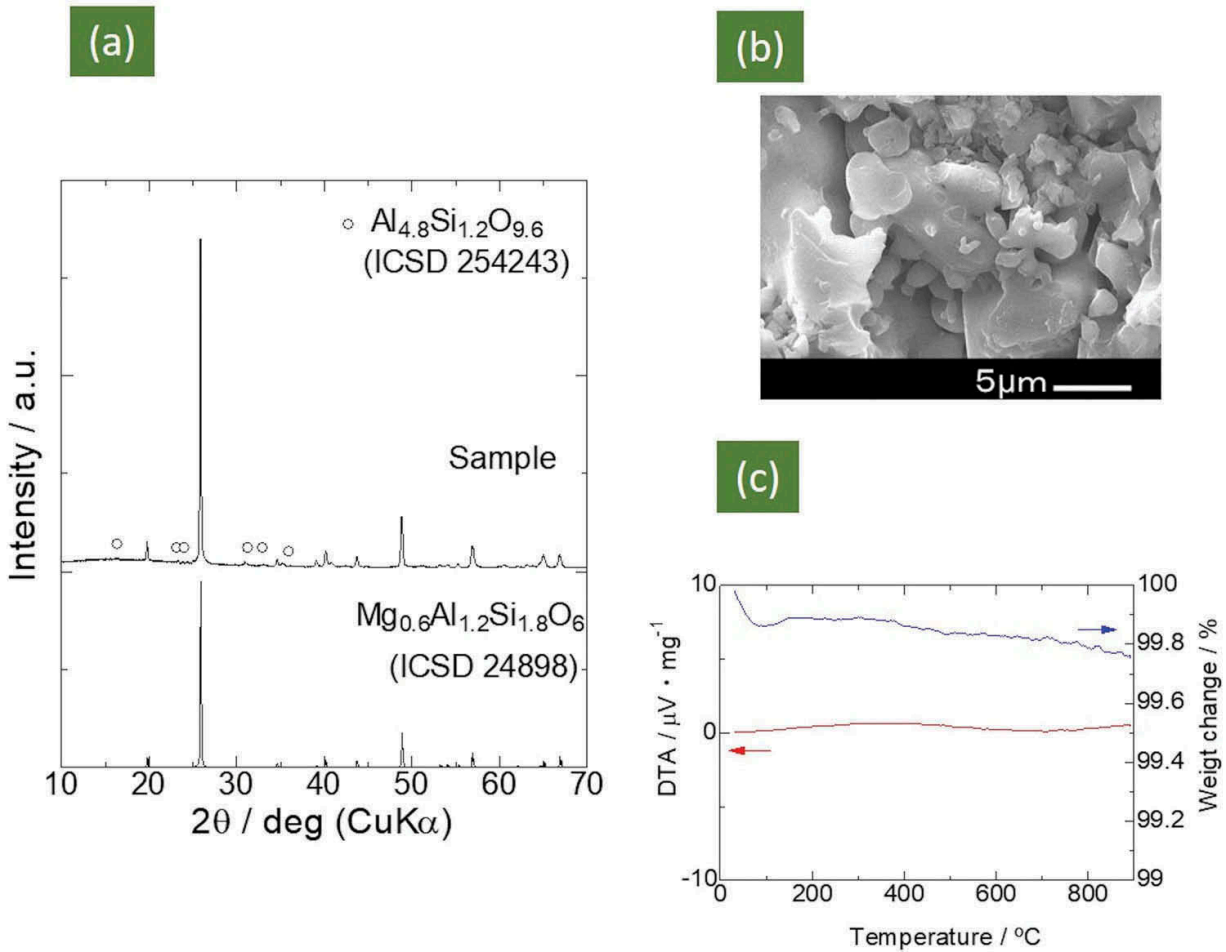
(a) XRD patterns of the Mg_0.6_Al_1.2_Si_1.8_O_6_ compound synthesized in this study and patterns obtained from ICSD datasets for comparison. The weak peaks indicated by open circles are ascribed to Al_4.8_Si_1.2_O_9.6_. (b) SEM image of sintered Mg_0.6_Al_1.2_Si_1.8_O_6_ sample. (c) TG-DTA curves of Mg_0.6_Al_1.2_Si_1.8_O_6_ compound

The XRD pattern of the sample calcinated at 700°C for 2 h and subsequently sintered at 1000°C for 2 h is shown in Figure 3(a). The formation of μ-cordierite Mg_0.6_Al_1.2_Si_1.8_O_6_ [25] (ICSD #24898) was confirmed, alongside a very small amount of the impurity phase of Al_4.8_Si_1.2_O_9.6_ (ICSD #254253). The relative density, i.e. the ratio of the apparent density of the sintered pellet to the density derived from the crystal structure (the true density), was 81%. Though we tried to improve the relative density by increasing the sintering temperature, another phase of cristobalite [ICDD: 01-080-3767] appeared after sintering at 1050°C (50°C above the synthesis temperature).

Figure 3(b) displays an SEM image of the fractured surface of the Mg_0.6_Al_1.2_Si_1.8_O_6_ sample sintered at 1000°C. Although there were pores of various sizes in the Mg_0.6_Al_1.2_Si_1.8_O_6_ sample, most of the particles were tightly connected. Hence, the pellet sintered at 1000°C (81% relative density) was used for the conductivity measurements discussed below. Figure 3(c) displays the TG-DTA curve; no marked change was observed in either weight or heat flow from 100°C to 1000°C (a small decrease in sample weight (~ 0.15% loss) below 100°C could be ascribed to the dehydration of adsorbed water.) Thus, proton contamination or oxygen vacancy formation in the solid state is unlikely, indicating that there would have been no proton or oxide ion conduction.

### 4.3. AC impedance measurements

**Figure 4.**
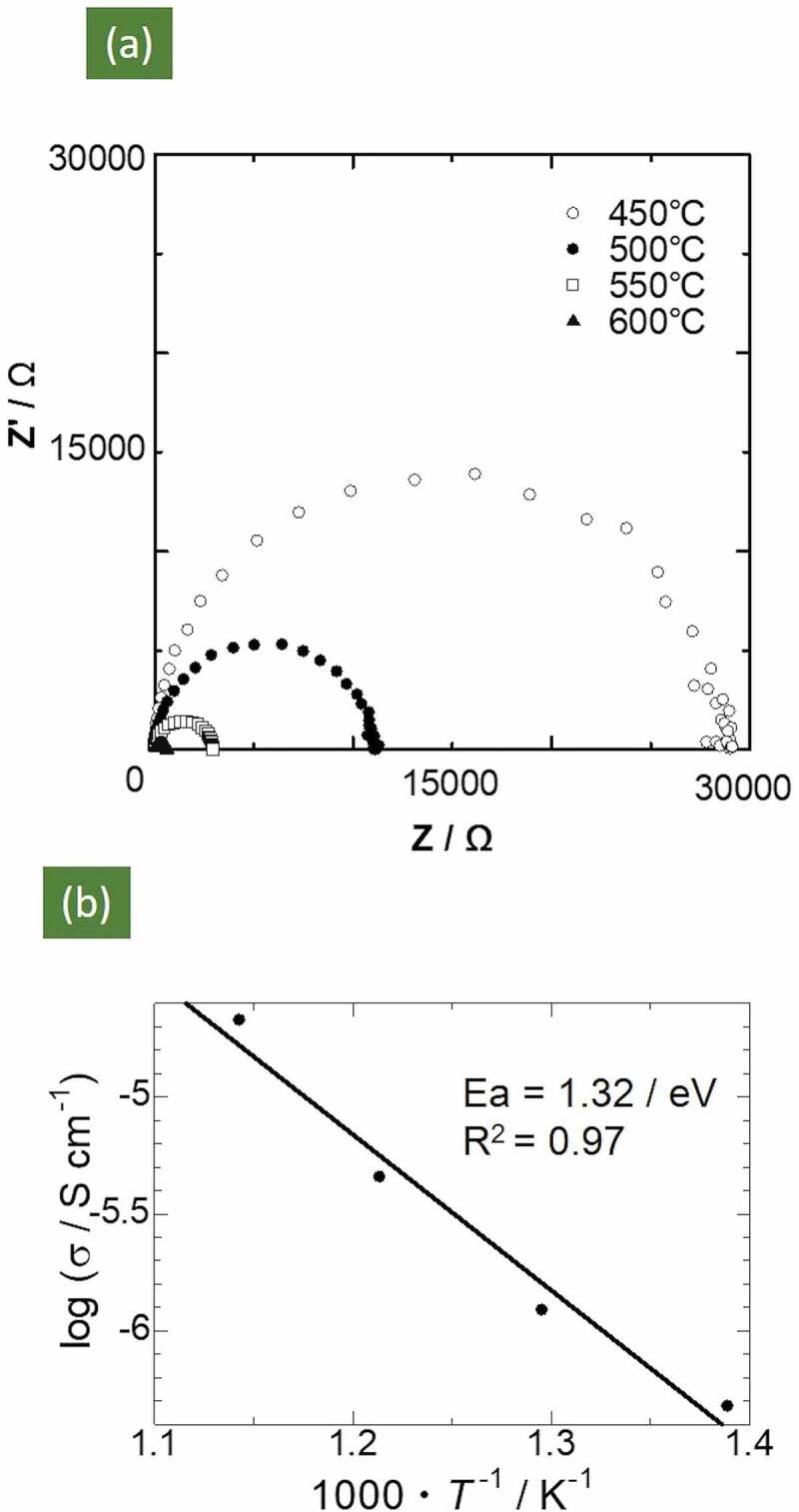
(a) Complex AC impedance spectra of Mg_0.6_Al_1.2_Si_1.8_O_6_ at various temperatures. (b) Arrhenius plots of ionic conductivity

The complex AC impedance plot of the Mg_0.6_Al_1.2_Si_1.8_O_6_ sample tested at 450–600°C is shown in Figure 4(a). The electrical resistance was calculated from the intercept on the Z’ axis. Plots in the form of single semicircles were obtained under all measurement conditions from 450 to 600°C, as shown in Figure 4(a). Therefore, we inferred that both the bulk and grain boundary resistances were merged in the semicircle, as the capacitance values determined from these semicircles were ~ 10^−10^ F, which lies in between both of these resistances. The impedance at the electrolyte | electrode interface was not observed; this may be identified in a lower frequency region than that used in this study. At 600°C, the electrical resistivity and conductivity calculated from the resistance values were 3.6 × 10^4^ Ωcm and 2.8 × 10^−5^ Scm^−1,^ respectively. The corresponding DC conductivity was 5.9 × 10^−9^ Scm^−1^ at 600°C, four orders of magnitude lower than the AC conductivity. This indicated that the mobile carriers were ions and not electrons.

The Arrhenius plot of the ionic conductivity obtained using the AC impedance measurements for the Mg_0.6_Al_1.2_Si_1.8_O_6_ sample is shown in Figure 4(b). A linear relationship is visible, and the evaluated activation energy was 1.32 eV. This result deviates from that obtained via FPMD calculations (0.95 eV). This deviation could be attributed to the contribution of the grain boundary resistance arising from the low relative density, as mentioned above.

### 4.4. Discussion

Table 1 summarizes the ionic conductivities of Mg^2+^ at 500°C in the present Mg_0.6_Al_1.2_Si_1.8_O_6_ compound (experimental and computational), alongside the representative compounds MgZr_4_(PO_4_)_6_ and Mg_0.7_ (Zr_0.85_Nb_0.15_)_4_ (PO_4_)_6_ for comparison. As mentioned earlier, the experimentally measured resistivity of Mg_0.6_Al_1.2_Si_1.8_O_6_ likely includes grain boundary resistance, and the ionic conductivity of this material can be increased by improving the sintering density. In addition, the ionic conductivity of Mg_0.6_Al_1.2_Si_1.8_O_6_ at 500°C was comparable to that of MgZr(PO_4_)_6_, which is a known fast Mg-ion conductor. The compositional optimization of Mg_0.6_Al_1.2_Si_1.8_O_6_, for example by doping or controlling the Mg:Al:Si molar ratio, may significantly improve the conductivity of Mg^2+^, as observed when Zr was partially replaced with Nb in MgZr_4_(PO_4_)_6_ (see Table 1). Note that the ionic conductivity of Mg_0.7_ (Zr_0.85_Nb_0.15_)_4_ (PO_4_)_6_ was two orders of magnitude higher than that of MgZr_4_(PO_4_)_6_.

Therefore, the high-throughput computational exploration partly succeeded in screening the Mg^2+^ conductive oxides, though the evaluated migration energies obtained by BVFF calculations deviated significantly from those obtained by DFT-MD studies and experimental observations for Mg_0.6_Al_1.2_Si_1.8_O_6_. We infer that the present BVFF calculations may systematically overestimate the migration energy, as our previous study for Li ion conductors confirmed systematic overestimation compared to DFT-derived migration energies [23]. The trend of conduction values may be captured even for Mg-O systems, and quantitative improvement is required for the present high-throughput scheme.

Materials knowledge is also useful when searching for Mg ionic conductors, as well as performing a high-throughput exhaustive search. To date, various materials design guidelines have been suggested for developing ion conductors. For example, the selection of anions with high polarizability [5] and/or anion packing structures [41,42] has been discussed. Other factors are related to the migration pathways in terms of changes in local coordination environments [18] or energy landscapes for traveling ions [43]. This well-considered physicochemical knowledge is indeed useful, though some of these guidelines are difficult to apply when screening thousands of compounds. It may be that combining this knowledge with present high-throughput computations, which suffer from deviations from DFT or experimentally observed ion conduction performances, could result in a more robust and rigorous scheme for finding new compounds.

## 5. Conclusions

High-throughput computational searches for Mg-ion conductors were performed, and their effectiveness was confirmed via FPMD calculations and experimental AC impedance measurements, to discover novel Mg^2+^ conductors. Mg_0.6_Al_1.2_Si_1.8_O_6_ with a μ-cordierite structure composed only of abundant elements, was identified as a fast Mg-ion conductor. However, the migration energies determined from the FPMD calculations and AC impedance measurements showed considerable deviation; this could be because of errors in setting the FF parameters or improper assumptions made in the high-throughput algorithm. Nevertheless, the experimentally measured ionic conductivity of Mg was comparable to that of MgZr_4_(PO_4_)_6_, a known fast Mg-ion conductor. Thus, the μ-cordierite Mg_0.6_Al_1.2_Si_1.8_O_6_ compound may show significant Mg-ion conductivity, after improving its sintering density and/or through composition optimization. This also implies that improving the robustness of the screening algorithm would accelerate the discovery of novel materials with fast ionic conductivity, compared to traditional trial-and-error or intuitive search methods.
